# Papillary Reconstruction using Hyaluronic Acid: A Review

**DOI:** 10.3290/j.ohpd.b3556037

**Published:** 2022-11-08

**Authors:** Thibault Lieppe, Charles Alliot, Christian Verner, Zahi Badran, Assem Soueidan, Xavier Struillou

**Affiliations:** a Assistant Professor, Department of Periodontology, Faculty of Dental Surgery, University of Nantes, Nantes, France. Idea, hypothesis, wrote the manuscript.; b Assistant Professor, Department of Periodontology, Faculty of Dental Surgery, University of Nantes, Nantes, France. Idea, hypothesis, wrote the manuscript.; c Associate Professor, Department of Periodontology, Faculty of Dental Surgery, University of Nantes, Nantes, France. Proofread the manuscript, contributed substantially to discussion.; d Professor, Department of Periodontology, Faculty of Dental Surgery, University of Nantes, Nantes, France; Periodontology Unit, College of Dental Medecine, University of Sharjah, Sharjah, UAE. Contributed to translation, critical review of the manuscript, elucidated biological function of hyaluronic acid.; e Professor, Inserm, UMR 1229, RMeS, Regenerative Medicine and Skeleton, University of Nantes, ONIRIS, Nantes, France; Department of Periodontology, Faculty of Dental Surgery, University of Nantes, Nantes, France. Proofread the manuscript, contributed substantially to discussion.; f Associate Professor, Inserm, UMR 1229, RMeS, Regenerative Medicine and Skeleton, University of Nantes, ONIRIS, Nantes, France; Department of Periodontology, Faculty of Dental Surgery, University of Nantes, Nantes, France. Idea, hypothesis, proofread the manuscript, contributed substantially to discussion.

**Keywords:** gingival papilla, hyaluronic acid, periodontal plastic surgery, periodontitis

## Abstract

**Purpose:**

The aim of this review is to analyse the use of hyaluronic acid injection in the interdental space to reconstruct the papilla in animal models and humans.

**Materials and Methods:**

Electronic databases were searched up to May 2022, and additional hand searching was performed. The search strategy was implemented according to the PRISMA guidelines. The inclusion criteria were: studies written in English, studies using hyaluronic acid, in vivo studies, studies with a precise number of specimens, case series with ≥6 patients, and studies published after 2010. The risk of bias was assessed for each study that could be evaluated.

**Results:**

A total of 19 articles were selected and reviewed in this review. Due to the great heterogeneity of the protocols and materials, comparison between studies was not possible. However, using this technique, the studies found statistically significant improvements in most cases. The filling percentages ranged from 19% to 100%. Regarding patient satisfaction, the few studies that evaluated this parameter found statistically significant results with most patients willing to repeat the experience. Regarding side effects, only two studies reported them. Moreover, the procedure does not seem to be very painful.

**Conclusion:**

The results suggest that hyaluronic acid injections seem to be effective in reconstructing papillary volume. However, to date, too few clinical trials with a high level of proof have been conducted on this technique. Future studies will have to work on the size of the sample, the concentration of the product, the number of injections and the injection method.

Since the advent of modern dentistry, interdental spaces in anterior aesthetics have become one of the most important topics. The interdental papilla is the gingival area that occupies the interproximal zone below the contact point between two adjacent teeth. The criteria for successful soft tissue management in the anterior region are largely based on the existence of a healthy interdental papilla and the formation of a suitable gingival contour.^9^

In addition to having a positive effect on smile aesthetics, interdental papillae are important in preventing food impaction between teeth and contribute to improved phonetics.

Therefore, a deficient interdental papilla, also known as a black triangle, can lead to pronunciation problems, food impaction, and aesthetic issues.^37^ Black triangles have been ranked as the third worst aesthetic problem, after cavities and poor crown margins.^12^

The black triangle problem between teeth due to a deficient papilla can result from several factors,^8,10,42^ e.g. the age of the subject, the shape/size of teeth, the length of the proximal contact point, and the interproximal gingival thickness. However, the most important factor seems to be increased distance from the contact point to the crest of bone.^8,45^

Different treatment options are often proposed to close an open gingival embrasure, such as orthodontics or restorative and prosthetic care.^17,20,23,37,39^ However, these are rather time-consuming and/or expensive procedures.

Some techniques have been developed in recent years to treat interdental papillae loss, such as surgery,^24,31,37^ which is considered to be unpredictable and risky, tissue engineering methods, such as the use of an injectable regenerative acellular dermal matrix,^16^ and autologous fibroblast injections.^30^

In 2010, Becker et al^4^ were the first to publish the idea of a non-surgical method in which the clinician injects a hyaluronic acid (HA) gel into the papilla to reconstruct its volume.

Hyaluronic acid is a member of the glycosaminoglycan family and a major component of the extracellular matrix in almost all tissues responsible for tissue resilience and volume due to its high hygroscopicity.^43^ Its use is increasing, particularly in cosmetic medicine.

Consequently, many authors have shown interest in this technique, including Mansouri et al,^38^ Awartani et al,^3^ and Lee et al,^26,27^ who described statistically significant improvements in clinical parameters after hyaluronic acid injection. For example, according to Mansouri et al,^38^ at 6 months, 43% of samples showed 50% or more improvement in interdental papilla reconstruction.

In this context, a non-surgical, minimally invasive, simple and relatively inexpensive technique of reconstruction of interdental papillae by HA injection appears to be an attractive therapeutic approach. It would probably be associated with less morbidity and few complications. In addition, it would generally be more cost-effective than invasive surgical techniques.

Thus, this study aimed to perform a review of pre-clinical and clinical studies on the use of hyaluronic acid injection in the interdental papilla with the intention of reconstructing it.

## Materials and Methods

### Study Registration

The review protocol was registered at the beginning of this work under the identification number CRD42021288768 in the PROSPERO international prospective register of reviews hosted by the National Institute for Health Research (NIHR), University of York, UK, Center for Reviews and Dissemination.

### Question

Based on the PRISMA directives^46^ (Preferred Reporting Items For Systematic Reviews and Meta-Analyses), a specific question was developed with the PICO (Participant, Interventions, Control, Outcomes) method:^13^ “Could hyaluronic acid injection improve the reconstruction of interdental papillae in humans and animals?”

### Information Sources and Search Strategy

The research strategy was developed according to the Preferred Reporting Items for Systematic Reviews and Meta-Analyses (PRISMA) guidelines as much as possible. Original articles were searched using electronic and manual databases from January 2010 until May 2022. Furthermore, relevant articles were screened by hand to potentially add relevant new articles. This search was applied to Medline, Cochrane Library, Google Scholar and Science Direct databases. The following Medical Subject Heading (MeSH) terms and keywords were used: “hyaluronic Acid OR hyaluronan” AND “papilla OR interdental papilla OR black triangles OR open gingival embrasure”.

Only articles in English were included, and no publication dates or publication status restrictions were imposed.

### Study Selection and Inclusion/Exclusion Criteria

**Fig 1:**
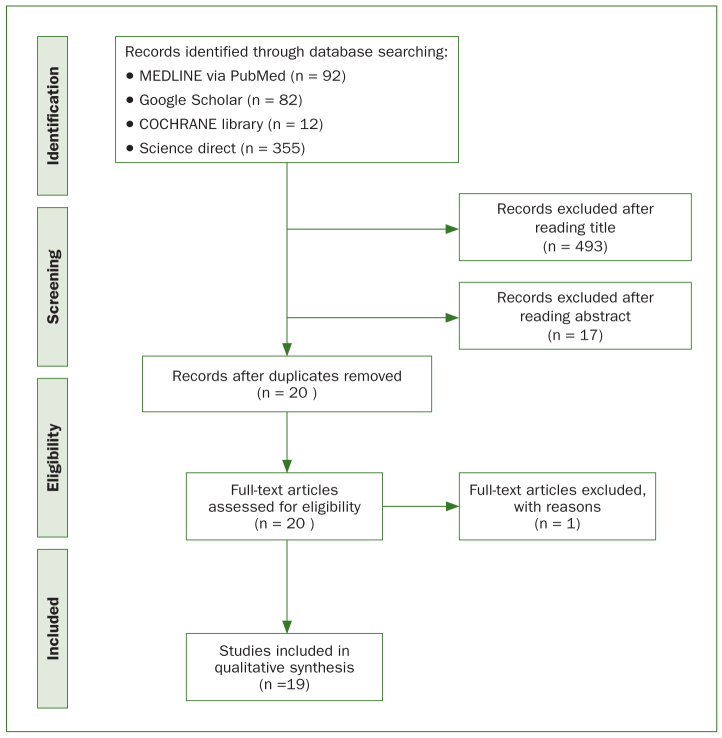
PRISMA flow chart

For the selection of studies, two investigators (TL, CA) screened the titles and the abstracts of the publications in a non-blinded, standardised manner. Selection was based on the inclusion and exclusion criteria defined to include only the most valuable articles (Table 1). Studies deemed to meet the inclusion criteria and those with insufficient information to make a clear decision were selected. The second phase consisted of the same investigators assessing the whole articles to determine eligibility for the study. The selection process was recorded in detail in a PRISMA flow diagram (Fig 1). Any disagreements between the two investigators regarding inclusion of a study were resolved by discussion.

**Table 1:** Inclusion and exclusion criteria

Inclusion criteria	Exclusion criteria
Studies written in English	In-vitro studies
Studies using hyaluronic acid	Retrospective studies
In-vivo studies, pre-clinical and clinical studies	Studies without statistical analysis
Studies with precise number of specimens	Reviews
Case series with ≥6 patients	Case reports
Studies published after 2010	Case series with <6 patients

### Data Collection Process and Data Items

The characteristics of the study were extracted independently by the same investigators and recorded. The data were compared for accuracy and any discrepancies were discussed and resolved by consensus.

Both reviewers extracted the following data from the included studies: 1. type of study; 2. cases; 3. observation period; 4. treated sites and classification used; 5. drop-out rate; 6. biomaterials; 7. injection method; 8. measurement method; 9. results; 10. adverse reaction (Table 2 and 3).

**Table 2:** Comparative table of pre-clinical animal studies on the use of hyaluronic acid

Reference	Type of study	Cases	Observation period	Treated sites and classification used	Drop out	Biomaterials	Injection method	Measurement method	Results	Adverse reaction
Pi S et al 2017^35^	Preclinical study	20 female rats (Orientbio, Seongnam, Korea) after 7 days of space opening with a spring	At days 0 and 2	Open gingival embrasure is standardised between the mandibular incisors after 7 days.	No	Restylane (a non-cohesive and cross-linked HA filler of non-animal origin)	Method: 20 μl of either PBS (n=10) or HA fillers (n=10) were locally injected into the interdental papilla, approximately 5-6 mm apical from the interdental papilla crest, using a 31-G needle. Frequency: once	Standardised serial photographs: At day 0 before the injection and at day 2 interdental distance and SPD (spring papilla distance) was evaluated. Micro CT was taken 2 days after the injection of PBS or HA filler. BPD (bone papilla distance) was evaluated. Histological examination thickness of 4 μm	Standardised serial photographs: The interdental distance was comparable in both PBS and HA filler groups. SPD was statistically significantly attenuated following the injection of HA filler compared to the injection of PBS (p<0.05). Micro CT: BPD was statistically significantly increased after the injection of HA filler compared to PBS (p<0.05). Histological examination: the interdental papilla became convex and inner granules containing hyaluronic acid were detected within the submucosal layer.	None
Kim SB et al 2020^22^	Preclinical study	20 male mice (Orientbio, Seongnam, Korea) after 5 days of space opening with a spring	At days 0, 2 and 7 post-injection	Open gingival embrasure is standardised between the mandibular incisors after 5 days.	No	Restylane	Method: 10 μl of either PBS (n=10) or HA fillers (n=10) was injected 1–2 mm below the IDP crest after upward the bevel of the syringe with a 30-G needle. Frequency: once	Standardised serial photographs: At day 0 before the injection, at days 2, 4 and 7. SPD (spring papilla distance) was evaluated. Histological analysis of IDP was performed using H&E staining on days 2 and 7 post-PBS or HA filler injection. Immunohistochemical analysis was performed to determine the localisation patterns of TNF-α, interleukin (IL)-1β, IL-6, myeloperoxidase (MPO), and Ki67.	Morphological examination: The SPD of the HA filler injection group was statistically significantly lower than that of the PBS injection group on days 2, 4, and 7 post-injection Histological analysis: HA filler was stable in the connective tissue underlying the epithelial tissue even on day 7 post-injection. Immunohistochemical analysis: TNF-α, IL-1β, IL-6, MPO, and Ki67 were highly localised to the connective tissue surrounding the filler on day 2, which decreased on day 7 post-injection.	None

**Table 3:** Comparative table of human studies in the use of hyaluronic acid

Reference	Type of study	Cases	Observation period	Treated sites and classification used	Number of patients excluded	Biomaterials	Injection method	Measurement method	Results	Adverse reaction
Abdelraouf SA et al 2019^1^	RCT	10 patients (3 males and 7 females) aged 21 to 47 years	At baseline, 3 months and 6 months	36 deficient interdental papillae (DIP) in the anterior region. 2 dropouts, thus 30 DIP HY group (n=16 papillae) Saline group (n=14 papillae) Papillary deficiency types I or II (Nordland and Tarnow classification)	2 patients excluded	Restylane Lidocaine (concentration of 20 mg/ml)	Method: 0.1 mm of HA gel or saline solution using a 30-gauge disposable insulin syringe was inserted 2–3 mm apical to the tip of the interdental papilla and directed coronally with an angulation of 45° to the long axis of the tooth, and the bevel directed apically. Frequency: at baseline, 3- and 6-weeks intervals	Clinical measurement: Height of the black triangle: distance between the deficient papilla tip and contact area (PT-CP distance) (probe+stent) Standardised digital clinical photographs: surface area of black triangles (SABT) (0.5 x height x base)	After three and six months from baseline: the results revealed a statistically significantly higher mean decrease in height and surface area of black triangles in favour of the HA group. From three to six months: there was no statistically significant difference between the two groups in both parameters. At 6 months: the HA group showed a statistically significantly higher mean satisfaction score than the saline group.	Not detected
Bertl K et al 2017^5^	RCT	22 patients (9 males, 12 females) mean age 36.4 years HA group (n=11) Saline group (n=10)	At baseline, 3 months and 6 months	21 deficient interdental papillae (DIP) in the anterior maxilla Modified papilla index score was used (MPIS)	1 patient excluded because of a crown fracture	Hyadent Barrier Gel (1 ml of the gel contains 16 mg cross-linked Na-hyaluronate and 2 mg Na-hyaluronate)	Method: Using pressure syringe for standardised dose delivery (0.06 ml per “click”) with a 30-gauge needle and a 3-step technique: 1. Creation of a reservoir in the mucosa immediately above the mucogingival junction (total amount ca. 0.18 ml) 2. Injection into the attached gingiva/mucosa just below the base of the deficient papilla (total amount ca. 0.12 ml) 3.Injection 2–3 mm apically to the tip of the deficient papilla (total amount ca. 0.06 ml) Frequency: At baseline then repeat at 4 weeks.	Clinical measurement: Width of KT, gingival phenotype, tissue texture and colour, MPIS, distance between the papilla tip (PT) and the contact point (CP), pocket depth (PD), clinical attachment level (CAL), bleeding on probing (BoP), and plaque. Standardized digital clinical photographs: Area of the “black triangle”. Intraoral scanner: Mucosal volume gain apically to the deficient papilla. Radiographic examination: Bone level measurement. Visual analogue scale (VAS): Pain level and aesthetic appearance.	No differences were observed between groups, neither at baseline nor at 3- and 6-months post-treatment. Mean PT-CP ranged between 1.8 mm and 2.3 mm without significant differences between groups or over time within groups; MPIS was 2 for all patients at all time points. Similarly, insignificant differences between groups or time points were observed for deficient area, gingival volume changes, bone level, and aesthetic appearance.	2 patients: discomfort, swelling, extreme tenderness with a burning sensation, skin discoloration The symptoms lasted up to 7 days, and in both cases, symptoms resolved without problems
Awartani FA et al 2016^3^	Case series	10 female patients mean age of 36.4 years (age range 22–55 years)	At baseline, 3 months and 6 months	17 anterior sites (13 maxillary, 4 mandibular) with DIP. 4 maxillary sites were classified as class II and 13 sites were classified as class I (Nordland and Tarnow classification)	1 excluded because of smoking	Hyadent Barrier Gel	Method: 0.2 ml Injected directly into the middle of the papilla, 2–3 mm apical to the tip of the papilla, using a 23-gauge needle. Frequency: at baseline, 21 days and then at 42 days.	Digital clinical photographs standardised: surface area (in mm^2^) (0.5 x height x base) based on image analysis software. % reduction area = (baseline area − postoperative area) × 100/baseline area. Patient satisfaction: based on surveys.	The lost interdental papilla area at baseline and at the 3- and 6-month postoperative visits was 1.2 ± 1.8 mm^2^ (mean ± SD), 0.6 ± 0.9 mm^2^, and 0.7 ± 0.7 mm^2^, respectively. Differences between baseline and postoperative visits were statistically significant (p < 0.0001) No statistically significant difference (p > 0.12) between the two postoperative time points.	Limited swelling and tenderness at the injection site were reported and typically lasted for the first 2–3 postoperative days.
WP Lee et al 2016^27^	Case series	13 patients (6 males 7 females) mean age of 32 years (age range 27–35 years)	At baseline and 6 months	57 treated sites in the maxillary anterior region	No patients excluded	Teosyal puresense Global action Teoxane	Method: The needle was inserted at a 45° angle, 2–3 mm apical to the involved papilla in a single-point injection technique, and the bevel of the injection needle was applied sloping upward. Each involved papilla was injected with a total of 0.01 cc by injecting 0.002 cc of hyaluronic acid gel each time. Frequency: injection 5 times every 3 weeks.	Clinical photographs standardised and radiographic examination standardised: (using a device) 3 measurements of the BT area (BTA), BT height (BTH), and BT width (BTW) were taken from clinical photographs. Radiographic measurements of the contact point-bone crest (CP-BC) and the interproximal distance between roots (IDR) were undertaken. The interdental papilla reconstruction rate (IPRR) was calculated to find the percentage change of BTA.	All sites showed improvement between treatment examinations. Thirty-six sites had complete interdental papilla reconstruction and 21 sites showed improvement ranging from 19% to 96%. The CP-BC correlated with the IPRR. More specifically, when the CP-BC reached 6 mm, virtually complete interdental papilla reconstruction via injectable hyaluronic acid gel was achieved.	None
WP Lee et al 2016^26^	Case series	10 patients (4 male and 6 female) mean age of 32 years (from 27 to 35 years)	At baseline and 6 months	43 treated sites in the maxillary anterior region	No patients excluded	Teosyal puresense Global action Teoxane	Method: The needle was inserted at a 45° angle, 2–3 mm apical to the involved papilla in a single-point injection technique, and the bevel of the injection needle was applied sloping upward. Each involved papilla was injected with a total of 0.01 cc by injecting 0.002 cc of hyaluronic acid gel each time. Frequency: injection was done 5 times every 3 weeks.	Digital clinical photographs standardised: Three measurements of the BT area (BTA), BT height (BTH), and BT width (BTW) were taken for each of the acquired clinical photographs. The image analysis program was used for the measurements.	29 sites had complete papilla reconstruction and 14 sites improved from 39 to 96% of interdental papilla reconstruction rate. Complete interdental papilla reconstruction was performed when black triangle at initial examination had area of ≤0.25 mm^2^, height of ≤1 mm, or width of ≤0.5 mm.	None
Shawky et al 2017^40^	Case series	30 female patients (from 25 to 35 years)	At baseline, 3 weeks, 3 months, six months	40 DIP in the anterior maxilla were divided into 2 groups, one received HA filler and the other one received Radiesse, with hydroxylapatite (CaHA) fillers. Papilla Presence Index (PPI) was used.	No patients excluded	Group A: HA, HYALGAN (Sodium Hyaluronate) Group B: RADIESSE (calcium hydroxylapatite)	Group A: a 23-gauge needle was used to inject less than 0.2 ml of HYALGAN, 2-3 mm apical to the coronal tip of the involved papillae. Group B: 0.2 ml of a RADIESSE **/LIDOCAINE*** blend was injected 2-3 mm apical to the coronal tip of the papillae.	Clinical measurement: Papilla Presence Index (PPI), Papillary Marginal Gingival Index (PMGI), Probing Depth (PD) and Clinical Attachment Loss (CAL). Standardised digital clinical photographs: the height of the papilla was measured using Image J software.	Significant improvement occurred in both groups but this improvement was more prevalent in Radiesse group compared with HA group. There was a decline in the papillary height in the HA group after three and six months, while in Radiesse group more long-lasting effect was denoted.	Radiesse filler showed mild inflammation after 3 weeks in 3 patients, while in the HA group, inflammation was initially denoted in only 2 patients (after 6 months).
Singh S et al 2019^41^	Case series	10 patients 25–40 years old	At baseline, 1 month, 3 months, and 6 months	42 DIP into 3 groups: HA 1%: 16 sites HA 2%: 14 sites HA 5%: 12 sites Total 35 sites were followed up out of 42	2 patients excluded	HA was prepared without adding any cross-linked agents. (known to cause allergic reaction) Injectable HA was prepared from raw powder at the College of Pharmacy.	Method: Injection 2–3mm apical to the coronal tip of the papilla Frequency: Injection was repeated on 2nd and 3rd weeks.	Clinical measurement: from contact point to tip of the papilla using the fixed reference point of stent. Photographic analysis: distance between the reference point and the tip of papilla using Image J software. Changes in the area of interest was calculated. Pain with a numeric scale	Clinical measurement: 5% of HA showed highly significant enhancement (p=0.001) of 19.2%, 20.6% 18.2% at 1, 3, and 6 months respectively. 2% of HA showed no significant enhancement (p = 0.125) 1% of HA showed significant enhancement (p = 0.004) Photographic analysis: 5% of HA showed highly significant enhancement of 41%, 42.9%, and 39.8% at 1, 3, and 6 months, respectively. However, intergroup comparison showed non-significant improvement	No significant perception of pain in 1% and 2% at each injection, in 5%, there was pain perception at all 3 injections. No adverse reaction
Ni J et al 2019^32^	Case series	8 female participants, mean age 41.6 years (from 28 to 60 years)	At baseline, 3 months, 12 months	22 DIP in the anterior (maxillary or mandibular) region with class I or II of Tarnow and Nordland classification.	No patients excluded	QI sheng biological agent 16 mg/ml HA.	Method: Every site received 0.05 to 0.1 ml of 16 mg/ml hyaluronic acid gel at the base of the deficient papilla. Frequency: at baseline, 3 weeks and 6 weeks.	Photograph examination: Photographs were assessed by a software based on image pixels to measure the height of the papilla and the area of the papilla.	The height of the gingival papilla increased 0.311, 0.45, and 0.4 mm from baseline at 3, 6, and 12 months, respectively, after treatment (p<0.05), whereas the area of the black triangle was reduced by 0.31, 0.41, and 0.36 mm^2^ at the same time points (p<0.05).	None
Çankaya ZT et al 2020^6^	Case series	20 patients (10 male and 10 female) mean age of 34 years (range 24 to 44 years)	At baseline 3 months, 12 months, 24 months	200 DIP in consecutive papillae regions in both arches Modified papilla index score (MPIS) with a score of 2 were included	No patients excluded	Hyadent Barrier Gel	Method: Three equidistant injections (27G needle) were made to the region corresponding to the triangle base; one injection was made to the top of the triangle, and two injections were made to the area between these two points. (amount of injection was decided by checking the pressure and tissue colour). Frequency: After the first session, injections were applied to the same points at 3-week intervals. (2nd or 3rd injection)	Digital impression method: Area of interdental space percentage change in the area value over time	A significant improvement was determined in the area values of both arches at 3, 12, and 24 months compared to the baseline values. The filling percentage was found to be 55% at 3 months, 72% at 12 months, and 79% at 24 months. More quickly and at a higher level in the maxilla	None
Alhabashneh R et al 2020^2^	Case series	21 (7 male 14 female) mean age of 36.9 years (±9.16 years)	At baseline, 3 weeks, 3 months, and 6 months	86 treated DIP (58 sites in the maxilla and 28 sites in the mandible) Classification according to Nordland and Tarnow	No patients excluded	Hyadent Barrier Gel	Method: Three step technique 0.2 ml of hyaluronic acid was injected with the three-step technique into each papilla. Frequency: injections were repeated after 21 days.	Photograph examination: Black triangle height and the amount of papilla filled were measured using software.	The mean reduction of black triangle (BT) height was 0.17 mm (8% reduction) (p<0.001), at the 3 months interval there was a mean reduction of BT height of 0.83 mm (39% reduction) (p<0.001). At 6 months however, the reduction in BT height was 0.62 mm (29% reduction) (p<0.001).	None
Firkova et al 2020^15^	Case series	19 patients (range 23–72 years)	At baseline, 1 month, 3 months, and 6 months	57 treated DIP class I and class II of Nordland Tarnow classification were included	No patients excluded	Hyadent Barrier Gel	Method: HA was injected 2–3 mm apical to the papilla tip with a 23-gauge needle directed coronally. The volume of the injected gel was administered individually till whitening of the adjacent tissue was noticed. Then few droplets of HA were topically applied and massaged onto the treated area. Frequency: The injection was repeated after 20 days	Photograph examination: Photographs were assessed by software based on image pixels to measure the height of the papilla and the area of the papilla.	There was a statistically significant change in the papilla level and fill of the interproximal space, ranging from 59% papilla gain on the first month, 72% to the third month and 77% on 6 months. In total, a significant reduction in papilla height of 1.92 mm was observed between baseline and the sixth month following treatment.	None
Mandel et al 2020^28^	Case series	40 patients (30 females and 10 males) mean age 44.0 (±13.03) Flex Barrier: n=20 Revident: n=20	At baseline, one week, one month	160 DIP (98 treated sites and 62 control sites) Nordland and Tarnow Classification NB: at least one upper and one lower papillary defect was left untreated and served as a negative control	9 patients were excluded (8 did not attend the control appointment and 1 developed a herpetic lesion)	Reference material: Flex barrier gel: (2/3 cross-linked and 1/3 non-cross-linked HA) VS Revident: Moscow, Russia 1% HA	Method: “Three-Step Technique”: (1) injection of the gel with a 30-G needle along the mucogingival junction at the base of the papilla at 4–5 sites, creating depots of 0.1ml per site, (2) injection of the gel into the attached gingiva at the base of the papilla at 2-3 sites, creating depots of 0.1ml per site, (3) injection of the gel into the papilla 2–3 mm from its tip at one site, creating a depot of 0.1 ml. Frequency: once	Photograph examination: Photographs were assessed by a software based on image pixels to measure the size of the black triangle.	As a rule, we can state that the smaller the initial lesion, the greater the improvement in the reduction of the “black triangle” lesion. However, the long-term results are largely dependent on the patient’s individual interdental oral hygiene rather than on the type of hyaluronic acid gel	None
Kapoor et al 2020^21^	Case series	6 patient (4 females and 2 males) mean age 37.5 (range 20–61 years)	At baseline 3 weeks 3 months and 6 months	7 treated sites in the maxillary anterior teeth Nordland and Tarnow classification (class I and II)	All participants were followed-up	A commercially available hyaluronic-based gel (<0.2ml) 0.2%.	Method: Local anaesthesia and injection of 0.2ml of 0.2% HA 2–3 mm to the tip of the papilla. Frequency: 2nd and 3rd doses at 3 weeks and 3 months.	Clinical measurement: Measurements of the black triangle were done clinically from the tip of the papilla to the contact point of the associated teeth using the periodontal probe and stent as a reference.	There was a statistically significant difference observed at 3 months in all seven cases.	No complication hypersensitivity or allergy was noted.
Patil et al 2020^34^	Case series	8 patients (3 males and 5 females) mean age 32 (range 27–35 years)	At baseline and 3 weeks 3 months	14 treated DIP in the upper anterior region	None	A commercially available hyaluronic-based dermal filler (<0.2 ml)	Method: injected 2–3 mm apical to the tip of the papilla Frequency: Patients were seen 3 weeks after the initial treatment, and if the dark space remained another injection was applied. This sequence was repeated up to three times.	Photograph examination: Clinical photographic measurements of the black triangle area (BTA), black triangle height (BTH), and black triangle width (BTW) were undertaken. For the analysis of the effects of the injectable HA gel, the interdental papilla reconstruction rate (IPRR) was defined as the percentage of change in the initial BTA to the final BTA after 3 months.	After performing interdental papilla reconstruction using injectable HA gel, a mean IPRR of 89.25% was found with 2.57 injections, whereas BTA, BTH, and BTW showed a mean decreased of 0.25, 0.85, and 0.34 mm^2^, respectively.	None
Pitale U et al 2021^36^	Case series	7 patients (2 males 5 females) 25 sites mean age 30.96	At baseline, 3 months and 6 months	25 treated DI Nordland and Tarnow classification	No patients dropped out	A commercially available cross-linked mild type, 20 mg/ml concentrated, 400 μm was obtained from GENOSS	Method: injection was made 2–3 mm apical to the coronal tip of papillae with a 23G × 25 mm intraoral injection needle at a 45° angle. Frequency: once	Photograph examination: Photographic analysis was performed by using the Image analysis software-GNU Image Manipulation Program (GIMP)	At 3 months, 52% (13 of 25) had complete papillary coverage clinically but 12 of 25 with image analysis. At 6 months, clinical measurement and image analysis method showed the same results: 12 of 25 sites had complete papillary coverage and 13 of 25 had partial papillary coverage.	Not described

If any of these data are not reported in the table, it means that the information is not mentioned by the authors. The systematic reviews^7,14^ examined were not included in our study because they did not meet the search criteria.

### Risk of Bias in Individual Studies

To ascertain the risk of bias in eligible articles, the same investigators in a blind manner evaluated their methodology either by SYRCLE’s Risk of Bias tool^19^ for animal intervention studies, or by the Cochrane Collaboration’s tool^18^ for human randomised trials. Case series were excluded from the risk of bias analysis because of the absence of adapted methodology.

### Data Synthesis

A meta-analysis could not be performed due to the heterogeneity in protocols of the studies and the products used. Consequently, we conducted a descriptive analysis of the studies.

## Results

### Study Selection

Taking into account the previously defined criteria, 541 studies were initially identified (Fig 1). The electronic search of Medline, Cochrane Library, Google Scholar and Science Direct databases provided 541 articles, and after adjusting for duplicates and reading the title and/or the abstract, 20 studies remained. Out of these, only 1 was discarded,^4^ because after reviewing these 20 articles, this paper clearly did not meet the inclusion criteria (number of cases <6). A total of 19 articles were included in the review.

### Study Characteristics

The 19 articles were then classified in comparative tables (Tables 2 and 3). For each selected study, the most important results are presented in Table 2 for the pre-clinical animal models and om Table 3 for the human studies

Tables 2 and 3 show an important variety in the types of hyaluronic acid used. The main brands, each with a different formula, are: Restylane, Hyadent BG, Teosyal puresense Global action Teoxane, Hyalgan, Flex barrier, Revident.

The work began with an analysis of the type of population in each article. The present study includes 2 pre-clinical animal studies,^22,35^ one on 20 female rats and the other on 20 male mice. It also includes 2 randomised clinical trials on humans,^1,5^ respectively containing 10 patients/36 treated deficient interdental papillae (DIP) and 21 patients/21 treated DIP, as well as 15 case series on humans including between 6 and 40 patients and between 7 and 200 treated DIP.

Among these studies, we found heterogeneity in the classifications used to evaluate the papillary defect. However, the classification by Nordland and Tarnow^33^ is the most commonly used. The same is true for the measurements and measurement methods used: clinical measurements, non-standardised photographs, standardised photographs, radiography, radiography with markers, digital impressions.

After local anaesthesia, two injection techniques were mostly used, the first consisting of injecting HA 2-3 mm apically into the top of the interdental papilla with variations depending on the orientation of the bevel. This technique was found in 15 of the 19 pre-clinical and clinical studies. The second, named “three-step technique” consisted of:

1. creation of a reservoir in the mucosa immediately above the muco-gingival junction;
2. injection into the attached gingiva just below the base of the deficient papilla; and
3. injection 2–3 mm apically of the tip of the deficient papilla, found in 4 of the 19 studies, all of which used Hyadent BG (recommendations for use).

The frequency of injection of biomaterials varied greatly from one study to another. Some studies proposed only one injection, while others proposed up to 5 injections spaced 3 weeks apart.

The observation period for these cases was generally 6 months, except for two studies which ran 24 and 25 months.^4,6^

We completed our review with the analysis of the number of patients excluded in each study (6th column in Table 3) and with the analysis of possible side-effects found after injection in the long or short term. In general, few side effects were described. Bertl et al^5^ found two patients with varying degrees of swelling, hypersensitivity, burning sensation, and discolouration of the mucosa.

### Risk of Bias within Studies

**Fig 2:**
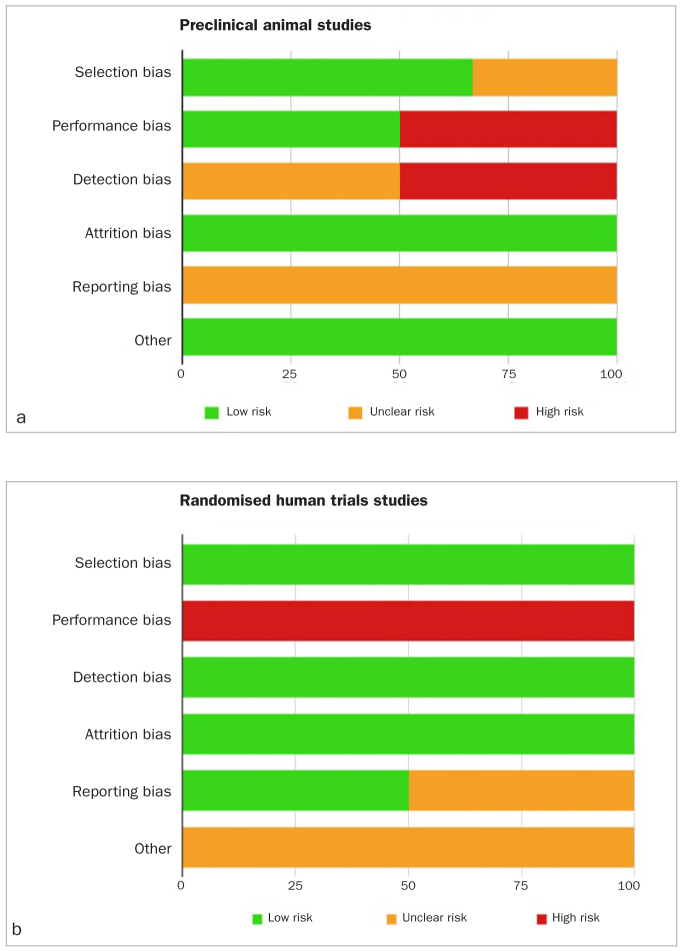
Risk of bias assessment of included studies. Risk of bias graph for animal studies, using the SYRCLE’s tool, averaged per item. Risk of bias graph for randomised human trials, using the Cochrane Collaboration’s tool, averaged per item. The green, yellow and red colours depict the percentages of studies with low, unclear or high risk of bias of the total number of assessed studies.

The results of the risk of bias assessment are shown in Fig 2. A suitable methodology was applied for each subgroup of studies: pre-clinical animal studies (Fig 2a) and randomised human trials (Fig 2b). Case series were excluded from the risk of bias analysis because their methods are not rigorous enough to carry out a proper risk of bias analysis.

Pre-clinical studies: Only 2 studies were included in this category. Our data show a high score of low risk of bias for selection bias (66%), attrition bias (100%) and other sources of bias (100%). However, a high score exists for unclear risk of bias for reporting (100%) and detection (50%) bias; and a high risk of bias concerning the blinding of these studies which were never mentioned.

Randomised human trials: Only 2 studies were includes in this category. Our data shows a high score of low risk of bias for selection (100%), detection (100%), attrition (100%), and reporting bias (50%). 50% of reporting bias and 100% of others sources of bias were uncleared after reading these studies. There is a high risk of bias concerning the performance bias because the clinicians in these studies were not blinded, whereas patients and the outcome assessor were. In fact, it was impossible for the researcher to be blinded due to the difference in consistency and resistance to injection between hyaluronic acid gel and saline solution.

## Discussion

Among all these studies, we noted important heterogeneity in the classifications used for papillary loss, in the biomaterials, and in the more or less standardised methods of measurement, making these studies difficult to analyse quantitatively. However, all agree on the aesthetic interest of this minimally invasive technique. Only anterior aesthetic regions (maxilla or mandible) were treated.

In the two preclinical studies,^22,35^ after having developed an animal model with deficient interdental papillae that is reliable and reproducible using a compression arch, the authors performed injections into these deficient papillae using hyaluronic acid. In these two studies of 20 animals each, significant decreases in the papilla-arch distance were found. Although these studies have biases and are difficult to translate to humans, the results are encouraging.

To date, relatively few studies on humans can be found in the literature. Only 2 randomised clinical trials^1,5^ representing the highest level of evidence could be found in this study. These trials are different in many ways: the biomaterial used, the method and frequency of injection, and the methods of measurement all differ.

The first trial, performed by Abdelraouf et al,^1^ resulted in a statistically significant decrease in the height and surface area of the black triangles in the hyaluronic acid group vs the saline group at 3 and 6 months compared to baseline.

This trial was performed only on natural teeth, unlike the Bertl et al^5^ study, in which the injection was performed only on papillae in maxillary anterior implants. Bertl et al^5^ found no difference between the groups (saline solution vs HA), either at 3 months or at 6 months, a result that contradicts Abdelraouf et al.^1^ This result could be explained by the differences in histological characteristics between the peri-implant mucosa and the gingiva around natural teeth. The peri-implant mucosa contains fewer fibroblasts than the gingiva and also presents reduced blood perfusion due to the absence of periodontal ligament.^11^ This reduced vascularity will limit the rheological effect of hyaluronic acid injected at the site.

Since the 2010 pilot study by Becker et al,^4^ which first described the innovative technique, several case series have been published. Of the 15 studies included in our review, all found statistically significant results. Although case series are of the lowest level of evidence and include major biases, the overall results tend to converge towards a decrease in the height and surface area of post-injection black triangles.

In the study by Singh et al,^41^ after comparison of different percentages of HA in injection, the results point towards the use of 5% HA, which would be more effective than 2% and 1% HA. In order to determine the ideal concentration to be injected, other studies with a larger number of patients and a longer follow-up must be conducted.

Shawky et al^40^ compared the effects of an alternative to HA, Radiesse, a product based on hydroxyapatite, which is increasingly used in cosmetic medicine. A statistically significant improvement was observed in both groups. However, the improvement was greater in the Radiesse group, which also had a longer-lasting effect but also slightly more inflammation. Further studies on the use of Radiesse in intra-papillary injection should be conducted to clarify whether Radiesse is clinically superior to hyaluronic acid gels. The aim of the present study was to establish a current overview of the techniques used to increase papillary volume; thus, we chose to include the Shawky et al study in order to examine more than one biomaterial. This allows us to see the other therapeutic options.

The best results of papillary reconstruction were obtained by Becker et al,^4^ with several successes of 100% reconstruction. The study by Abdelraouf et al,^1^ which is the only RCT at the dental level, obtained only 45% of average reconstruction. They explain this difference by the difference in the magnitude of the papilla loss included in the studies performed prior to theirs. Regarding the papillae around the implants, Bertl et al^4^ had positive results of up to 100% reconstruction.

Few studies have assessed patient satisfaction. In the study by Abdelraouf et al,^1^ a statistically significant difference was found at 6 months in favour of the group which received hyaluronic acid. Awartani et al^3^ stated that two-thirds of their patients found this new protocol satisfactory. Finally, Firkova et al^15^ found that the procedure was very well tolerated by patients, who reported it to be not painful. Most of their patients were very satisfied with the achieved aesthetic results.

In 2020, Ficho et al^14^ published a systematic review of the literature on the same subject, finding that the percentage of papillary reconstruction after 6 months of application is 77.4%, with an average number of applications of 3.17. However, with their chosen criteria and in order to obtain quantitative results, they could only include 4 case-series studies and their bibliographic search ended in in June 2019.

The latest systematic review of the literature published in April 2022 by Castro-Calderón,^7^ including 11 articles, propose a similar conclusion to our study: “Hyaluronic acid might be clinically used to re-create the interproximal lost papilla.”^7^ Our literature review provides a broader view of the use of this technique, with more articles included. We decided to include as many studies as possible in order to have the most global presentation possible of the current state of the literature on this technique. In all the publications studied, only that of Bertl et al^5^ reported undesirable effects, in addition to the mild post-operative pain already described.

Hyaluronic acid is currently the focus of much research in periodontics. Looking for different therapeutic applications in addition to its possible effects on the papillae, studies have also examined it potential role bone regeneration of periodontal defects.^25,29^

The limitations of this review are the small number of studies included and the low power of the studies themselves. In addition, with our objective of having a broad view of the technique, we included pre-clinical and animal studies. We wanted to keep them in our analysis in order to provide a comprehensive overview of what the literature currently offers on the subject.

At this time, this technique lacks a clear protocol and indications, so it is difficult to perform a rigorous methodological analysis on a small number of studies with different protocols. However, all the results tend to point towards success in terms of volume and satisfaction. Furthermore, the low number of adverse events suggests that this biomaterial has a bright future.

## Conclusion

Within the limitations of this study, the use of a commercially available hyaluronic acid gel for the treatment of interdental papillary deficiency seems to be effective and promising. In addition, a realistic prognosis must be presented to the patients regarding the expected results of treatment; the possibility of reinjecting HA into the deficient papillae in the longer term must also be explained to the patients. The indications must be very precisely defined and the patients well chosen.

Randomised clinical trials are needed in the longer term to obtain studies with a better level of proof to validate this technique and the various indications. Furthermore, the following factors must be address in future studies: the size of the sample for more power and representativeness, the concentration of the product, the number of injections and the injection technique. This will facilitate establishing a standardarised protocol and also respond adequately to the aesthetic requests of our patients.
